# Palliative radiotherapy in patients with a symptomatic pelvic mass of metastatic colorectal cancer

**DOI:** 10.1186/1748-717X-6-52

**Published:** 2011-05-21

**Authors:** Sun Hyun Bae, Won Park, Doo Ho Choi, Heerim Nam, Won Ki Kang, Young Suk Park, Joon Oh Park, Ho Kyung Chun, Woo Yong Lee, Seong Hyeon Yun, Hee Cheol Kim

**Affiliations:** 1 Department of Radiation Oncology, Samsung Medical Center, Sungkyunkwan University School of Medicine, Seoul, Korea; 2 Department of Radiation Oncology, Kangbuk Samsung Medical Center, Sungkyunkwan University School of Medicine, Seoul, Korea; 3 Department of Medicine (Division of Hematology/Oncology), Samsung Medical Center, Sungkyunkwan University School of Medicine, Seoul, Korea; 4 Department of General Surgery, Samsung Medical Center, Sungkyunkwan University School of Medicine, Seoul, Korea

**Keywords:** metastatic colorectal cancer, pelvic recurrence, palliative radiation therapy, concurrent chemoradiotherapy

## Abstract

**Background:**

To evaluate the palliative role of radiotherapy (RT) and define the effectiveness of chemotherapy combined with palliative RT (CCRT) in patients with a symptomatic pelvic mass of metastatic colorectal cancer.

**Methods:**

From August 1995 to December 2007, 80 patients with a symptomatic pelvic mass of metastatic colorectal cancer were treated with palliative RT at Samsung Medical Center. Initial presenting symptoms were pain (68 cases), bleeding (18 cases), and obstruction (nine cases). The pelvic mass originated from rectal cancer in 58 patients (73%) and from colon cancer in 22 patients (27%). Initially 72 patients (90%) were treated with surgery, including 64 complete local excisions; 77% in colon cancer and 81% in rectal cancer. The total RT dose ranged 8-60 Gy (median: 36 Gy) with 1.8-8 Gy per fraction. When the **α/β **for the tumor was assumed to be 10 Gy for the biologically equivalent dose (BED), the median RT dose was 46.8 Gy_10 _(14.4-78). Twenty one patients (26%) were treated with CCRT. Symptom palliation was assessed one month after the completion of RT.

**Results:**

Symptom palliation was achieved in 80% of the cases. During the median follow-up period of five months (1-44 months), 45% of the cases experienced reappearance of symptoms; the median symptom control duration was five months. Median survival after RT was six months. On univariate analysis, the only significant prognostic factor for symptom control duration was BED ≥40 Gy_10 _(p < 0.05), and CCRT was a marginally significant factor (p = 0.0644). On multivariate analysis, BED and CCRT were significant prognostic factors for symptom control duration (p < 0.05).

**Conclusions:**

RT was an effective palliation method in patients with a symptomatic pelvic mass of metastatic colorectal cancer. For improvement of symptom control rate and duration, a BED ≥ 40 Gy_10 _is recommended when possible. Considering the low morbidity and improved symptom palliation, CCRT might be considered in patients with good performance status.

## Background

Local recurrence of colorectal cancer after surgery occurred in 10-40% of patients [1-4]. Although local control has been improved with adjuvant chemotherapy and radiotherapy (RT), approximately 10-25% of patients still develop recurrence of disease in the pelvis [5-7]. Pelvic recurrence contributes significantly to the clinical course and is one of the major problems affecting the quality of life in these patients [8,9].

The role of primary tumor resection for non-curable stage IV colorectal cancer remains undefined. Kleespies et al. reported that palliative resection was associated with a particularly unfavorable outcome in rectal cancer patients presenting with locally advanced lesion expected macroscopic residual tumor or an extensive comorbidity [10]. Patients with prior curative resection of colorectal cancer often present with pelvic pain, one of the common manifestations of local recurrence involving nerves in the presacrum or pelvic sidewalls. The surgical approach to relieve symptomatic pain in the pelvis is usually unlikely to have negative resection margins [11]. Accordingly, resection of symptomatic pelvic tumor may be warranted only in those with adequate performance status and a resectable tumor burden with a possibly negative resection margin.

RT has been considered an effective palliative treatment for patients with symptomatic pelvic tumors of colorectal cancer. Previous studies have used RT as palliative treatment to relieve pelvic symptoms in heterogeneous patients [9,11-23]. These studies included patients with unresectable local recurrence after definitive surgery and symptomatic local recurrence with distant metastasis. Recently, modern combination chemotherapy including targeted drugs has been used to treat patients with metastatic colorectal cancer patients [24,25]. Although these drugs might be effective for symptomatic palliation in responding patients, the role of RT in addition to chemotherapeutic or targeted drugs for palliation is controversial. To date, there have been few reports of the synergistic effect of concurrent chemoradiotherapy (CCRT) with palliative intent in gastrointestinal cancer [22,26-30].

In this study, we evaluated the palliative role of RT and defined the effectiveness of chemotherapy combined with palliative RT in patients with a symptomatic pelvic mass of metastatic colorectal cancer.

## Methods

From August 1995 to December 2007, we retrospectively reviewed 80 patients with a symptomatic pelvic mass of metastatic colorectal cancer who were treated with palliative RT at Samsung Medical Center. All patients had disease outside the pelvis and consulted a radiation oncologist for symptomatic palliation.

Fifty-eight patients (73%) were initially diagnosed with rectal cancer and 22 patients (27%) with colon cancer. Seventy-two patients (90%) were treated with surgery, including 64 complete local excisions (52 curative surgeries). Eight patients (10%) received chemotherapy alone. Twenty-two patients (27%) received adjuvant RT after curative surgery or salvage RT after local recurrence. The RT dose ranged from 40Gy to 74Gy with 1.8-2.0 Gy per fraction.

Patient age at the time of palliative RT for symptomatic pelvic mass ranged from 27 to 85 years (median 57 years). There were 43 males (54%) and 37 females (46%). Eastern Cooperative Oncology Group (ECOG) performance scores were 0 in one patient (1%), 1 in 28 (35%), 2 in 36 (45%), 3 in 13 (16%), and 4 in two (3%) patients. Forty patients (50%) had single organ metastasis regardless of the number of metastatic nodules, and 40 patients (50%) had two or more organ metastases. The common metastatic sites were liver (33 patients, 41%), lung (29, 36%), paraaortic lymph node (27, 34%), and peritoneal seeding (15, 19%).

Eighty patients had 95 cases of clinical symptoms. The most common clinical symptom was pain (68, 72%), followed by rectal bleeding in 18 cases (19%), and obstructive symptoms in nine cases (9%). Six patients had two concurrent symptoms, pain and bleeding (five patients) or pain and obstruction (one patient). Seven patients experienced pain recurrence after palliative RT and received palliative re-RT. In these patients, evaluation of the response to palliative re-RT was available, and all cases were independently included in the analysis of symptom response to palliative RT.

RT was administered using 6-15 MV photon beams from linear accelerators. The radiation field included the recurrent mass of the pelvic cavity with 2-3 cm margins. Different RT doses were used according to the patient's performance status and extra-pelvic tumor burden. The median RT dose was 36 Gy (8-60 Gy). All patients received RT once daily, and the dose per fraction ranged from 1.8 Gy to 8.0 Gy (median 2.5 Gy). The total RT doses were converted into the biologically equivalent dose (BED) for comparison. When **α/β **was assumed to be 10 Gy, the median total BED was 46.8 Gy_10 _(14.4-78.0 Gy_10_).

Twenty-one patients (26%) received CCRT with the following regimens: capecitabine in eight patients, fluorouracil in six patients, oral tegafur-uracil in five patients, and combination regimen in two patients. Thirty-one cases received further chemotherapy after completion of palliative RT. Patient and treatment characteristics are shown in Table 1.

**Table 1:** Patient and treatment characteristics

Characteristic	No. of patients (%)
Age	Median: 57 years
< 60 years	43 (54)
≥ 60 years	37 (46)
Gender	
Male	43 (54)
Female	37 (46)
ECOG* performance status	
0-2	65 (81)
3-4	15 (19)
Primary site	
Colon cancer	22 (27)
Rectal cancer	58 (73)
Status of distant metastasis	
Single organ involved	40 (50)
Multiple organs involved	40 (50)
Symptom^b^	
Pain	68 (72)
Bleeding	18 (19)
Obstruction	9 (9)
Re-irradiation^†^	
Yes	30 (32)
No	65 (68)
Biologically equivalent dose^†^	
< 40 Gy_10_	34 (36)
≥ 40 Gy_10_	61 (64)
Concurrent chemotherapy^†^	
Yes	22 (23)
No	73 (77)
Adjuvant chemotherapy^†^	
Yes	31 (33)
No	64 (67)

* ECOG: Eastern Cooperative Oncology Group

^†^Treatment characteristics: 80 patients had 95 cases of clinical symptoms.

Symptom palliation was assessed one month after the completion of palliative RT. For patients with pain as the presenting symptom, effective palliation was defined as decreased or resolved pain or decreased analgesia. For patients with bleeding, effective palliation was defined as improved hemoglobin, stable hemoglobin, resolved hematochesia, or normalized hemoglobin. For patients with obstruction, effective palliation was defined as improved or resolved constipation, decreased laxative use, or no need for intervention such as a stent or colostomy.

The follow-up period was defined as the time from the start of palliative RT until progression of symptom or death. Toxicity was assessed according to the Common Terminology Criteria for Adverse Events (CTCAE) version 3.0.

Survival rates were estimated with the Kaplan-Meier method, and comparisons between the groups were determined using the log-rank test [31]. Multivariate analysis was performed to assess the relationships between the outcomes and possible prognostic variables using the Cox proportional hazards model [32]. The Chi-square test was used to analysis differences in patient and treatment characteristics between the symptom control group and the recurrent group. Statistical analyses were performed using SAS software (SAS for Windows, version 9.0, SAS Institute, Cary, NC, USA).

## Results

The median follow-up time was five months (range, 1-44 months). The overall survival rate at one year was 22.1%, and the median survival was six months. Overall symptomatic palliation was achieved in 76 of 95 cases (80%). Forty-three cases (45%) experienced recurrence of symptoms, and the median symptom control duration was five months (range, 1-44 months).

In 80 patients, 68 cases had the symptom of pain; 54 cases (79%) achieved palliation of pain and 35 cases (51%) experienced reappearance of pain. Eighteen cases had the symptom of bleeding; 15 cases (83%) achieved palliation of bleeding and five cases (28%) experienced reappearance of bleeding. Nine cases had the symptom of obstruction; seven cases (78%) achieved palliation of obstruction, and three cases (33%) experienced reappearance of obstruction.

**Figure 1:**
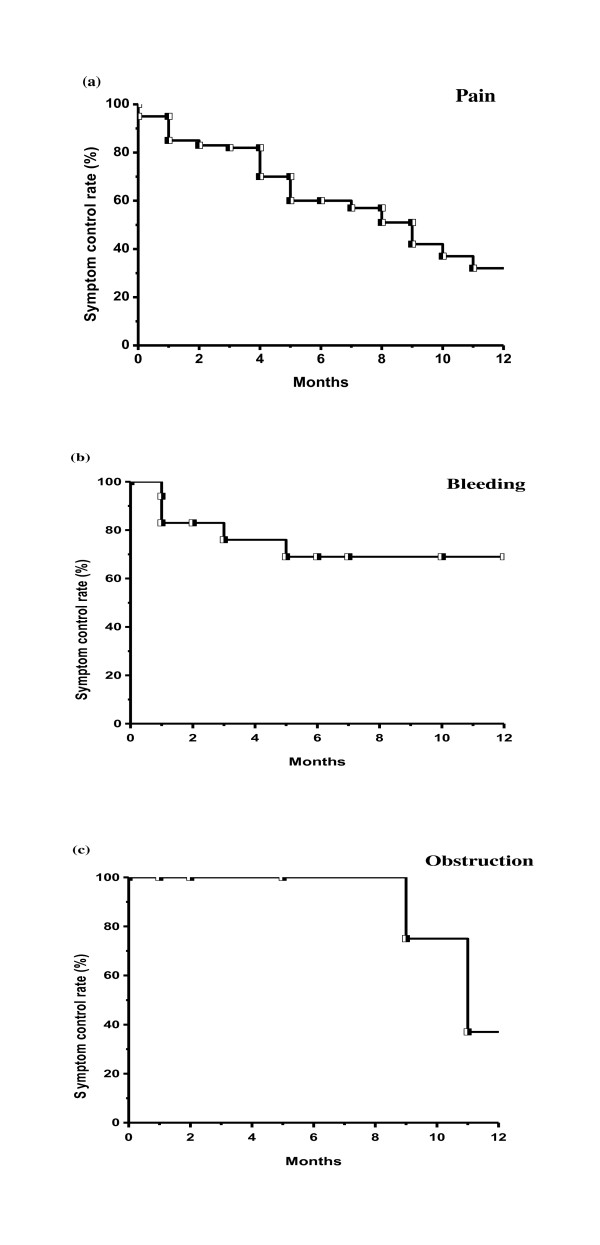
**Symptom control rates according to initial presenting symptom**. (a) pain, (b) bleeding, (c) obstruction.

Figure 1 shows symptom control rates according to initial presenting symptom. One-year symptomatic control rates were 32.1%, 69.9%, and 37.5% for pain, bleeding, and obstruction, respectively. Table 2 shows the recurrence rate after symptom palliation. BED was a statistically significant factor affecting recurrence after symptom palliation (p = 0.0011). For BED < 40 Gy_10, _23 of 34 cases (68%) had recurrence and for BED ≥ 40 Gy_10_, and 20 of 61 cases (33%) had recurrence.

**Table 2:** Symptom recurrences according to patient and treatment characteristics in patients with palliative symptom control after palliative treatment.

Characteristic	Total control number	Recurrence (%)	p-value
Symptom			
Pain	33 (49)	35 (51)	0.1499
Bleeding	13 (72)	5 (28)	
Obstruction	6 (67)	3 (33)	
Re-irradiation			
Yes	16 (53)	14 (47)	0.8519
No	36 (55)	29 (45)	
Biologically equivalent dose			
< 40 Gy_10_	11 (32)	23 (68)	0.0011
≥40 Gy_10_	41 (67)	20 (33)	
Concurrent chemotherapy			
Yes	14 (44)	8 (36)	0.3387
No	38 (52)	35 (48)	

**Figure 2:**
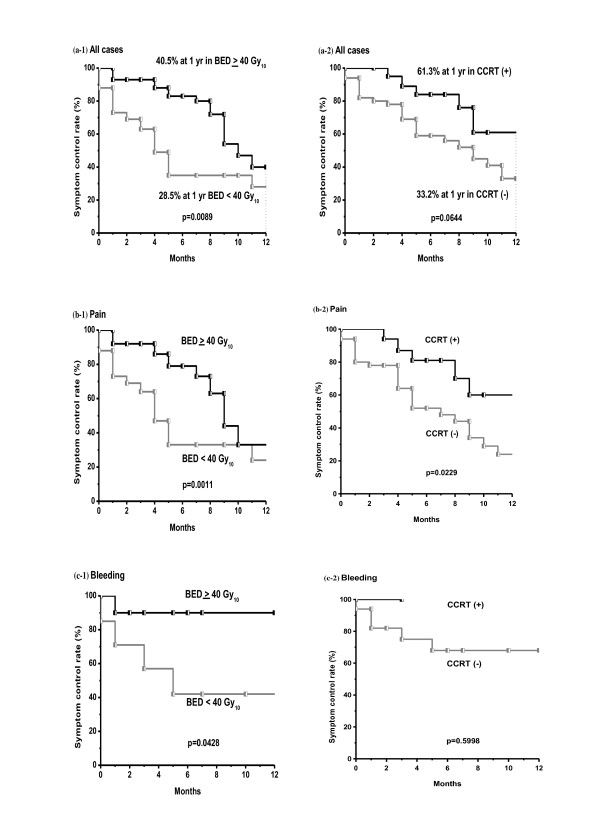
**Symptom control rates according to the biologically equivalent dose (BED) and concurrent chemotherapy during RT (CCRT)**. (a) BED more than 40 Gy_10 _was a statistically significant prognostic factor (p = 0.0089) on univariate analysis in all cases. CCRT was a marginally significant factor (p = 0.0644) on univariate analysis in all cases. (b) In patients with pain, higher BED and CCRT was associated with improved outcome with statistical significance. (c) In patients with bleeding, only higher BED was statistically significant factor.

On univariate analysis, the only significant prognostic factor for symptom control rate was BED (p = 0.0089), and CCRT was a marginally significant factor (p = 0.0644) (Table 3). In patients with pain, higher BED and CCRT was associated with improved outcome with statistical significance. In patients with bleeding, only higher BED was statistically significant factor. In nine patients with obstruction, there was no significant factor associated with symptom relief. Figure 2 shows differences in the symptom control rate according to BED and CCRT. Multivariate Cox regression analysis of prognostic factors for the symptom control rate revealed that higher RT dose (BED ≥ 40 Gy_10_, hazard ratio; 0.503, p = 0.0406) and CCRT (hazard ratio; 0.427, p = 0.0449) were favorable factors.

**Table 3:** Univariate analysis of factors affecting symptom control rate in 95 cases

Characteristic	One-year symptom control rate (%)	p-value
Age		
< 60 years	22.6	0.5121
≥ 60 years	46.8	
Gender		
Male	45.1	0.1210
Female	29.2	
ECOG* performance status		
0-2	36.2	0.8387
3-4	27.0	
Primary site		
Colon cancer	38.8	0.9967
Rectal cancer	37.8	
Symptom		
Pain	32.1	0.1029
Bleeding	69.9	
Obstruction	37.5	
Biologically equivalent dose		
< 40 Gy_10_	28.5	0.0089
≥ 40 Gy_10_	40.5	
Re-irradiation		
Yes	41.0	0.3750
No	36.5	
Concurrent chemotherapy		
Yes	61.3	0.0644
No	33.2	
Adjuvant chemotherapy		
Yes	32.2	0.1986
No	46.8	

* ECOG: Eastern Cooperative Oncology Group

All patients tolerated the palliative treatment. Thirty-eight patients experienced nausea, diarrhea, cystitis, or perineal skin reaction of grade 1 or 2 during treatment. There was no significant difference of treatment related toxicity between RT alone and CCRT (45% vs. 52%, p = 0.4380). There was no severe toxicity above grade 3 or treatment-related deaths.

## Discussion

External RT has been considered an effective palliative treatment in patients with an unresectable pelvic mass of colorectal cancer. Studies reported in the 1960s-1980s showed that relief of pain and/or bleeding was achieved in approximately 75% of patients with doses as low as 20 Gy in ten fractions over two weeks or various doses of 40-60Gy in 1.8-2.5 Gy per fraction [11-20]. Median duration of symptom relief was only 6-9 months. Later reports showed similar or improved results of symptom palliation: control of pain in 78-93% of patients, control of bleeding in 68-100%, and control of mass in 35-88% [9,21-23]. The rate and duration of symptom palliation in our study were similar to those of previous reports.

Some data suggest a correlation between RT dose and the effect of palliation. Wong CS et al. [9] reported the response rate of pelvic symptoms according to RT dose. There was a trend suggesting increased response rates with increasing total RT dose. For pain improvement after RT, 48% of patients responded after a total dose of less than 20 Gy, 77% responded after a dose of 20-30 Gy, 79% after 30-45 Gy, and 89% after 45 Gy or more. For residual, inoperable, or recurrent lesions, Wang CC and Schulz MD [17] reported that the percentage of patients with controlled symptoms for six months or more increased with dose (12% with 21-30 Gy, 31% with 31-40 Gy, and 58% with 41-50 Gy). Crane CH et al. [22] used hypofractionated RT with three different dose regimens (30 Gy/6 fractions, 35 Gy/14 fractions and 45 Gy/25 fractions). On univariate analysis, BED < 35 Gy_10 _showed a higher risk of pelvic symptomatic progression when **α/β **was assumed to be 10 Gy(p = 0.009). In our study, the overall symptom control rate and the one-year symptom control rate were 69% and 40.5% respectively for BED ≥ 40 Gy_10 _and 32% and 28.5% for BED < 40 Gy_10_. Since BED was a significant prognostic factor for symptom control rate, it might be better to treat with a higher RT dose to increase the palliation rate and symptom control duration.

In the pelvic cavity, the tolerance dose of normal tissue is a limiting factor when determining the RT dose. The small bowel, which is regarded as one of the most sensitive organs to RT, has a tolerance dose of TD 5/5 with a dose of 50 Gy for 1/3 small bowel irradiation and TD 50/5 with a dose of 60 Gy [33]. The risk of injury to the bowel is increased in cases with a history of previous surgery. However, there is evidence suggesting that significant recovery of the RT effect occurs with time [23,34]. Nieder C et al. [34] reported that acute responding tissues recovered from radiation injury within a few months and could then tolerate another full course of radiation. For late toxicity endpoints, the skin, mucosa, lung, and spinal cord do partially recover from subclinical injury at a magnitude dependent on the organ type, size of the initial dose, and, to a lesser extent, the interval between radiation courses. Mohiuddin M et al. [23] reported long term results of re-RT for patients with recurrent rectal cancer. They suggested a re-RT dose according to the interval between previous RT and re-RT as follows: 35 Gy for an interval of 3-12 months, 40-45 Gy for 12-24 months, 45-50 Gy for 24-36 months, and 50-55 Gy for more than 36 months. In our study, 27% of patients received re-RT, and there was no severe toxicity, including RT-induced fistula.

There are a few studies on CCRT for palliative intent in patients with distant metastasis. Wong CS et al. [9] reviewed 519 patients with locally recurrent rectal cancer treated with RT. Concurrent extrapelvic distant metastases were found in 164 patients. Twenty-two patients received CCRT with 5-fluorouracil and mitomycin C in a pilot study of combined modality therapy. Ten of the 22 patients were unable to complete the treatment protocol because of excessive acute hematological and gastrointestinal toxicity, and five patients developed neutropenic sepsis, one of whom died [35]. Crane CH et al. [22] first described the use of CCRT as an initial measure in 80 patients with synchronous distant metastasis from rectal cancer. Symptoms from the primary tumor resolved in 94% of cases, and progression occurred at a median of 33 weeks. There were acute complications of Radiation Therapy Oncology Group (RTOG) Grade 3 or greater in four patients, severe perioperative complications in five patients, and no significant late treatment-related complications. They concluded that initial pelvic CCRT produced high pelvic symptom control rates and that patients can be safely treated using this modality. In a study of the clinical benefit of palliative CCRT in advanced gastric cancer, 37 patients were treated with palliative RT (median dose 35 Gy) and nearly two-thirds of all patients received CCRT [26]. The overall symptom control rate was approximately 70%, which was superior to the previous 25-54% control rate for palliative RT alone [36]. Some studies reported the benefit of palliative CCRT for dysphagia in advanced esophageal cancer [27-30]. A phase I/II trial from Canada [29] prospectively treated 22 patients with dysphagia from advanced incurable esophageal cancer with palliative RT (30 Gy/10 Fractions) and a concurrent single course of chemotherapy (5-FU and mitomycin-C). Treatment was generally well tolerated and 68% achieved a complete response. The median dysphagia-free interval from time of onset of improvement was 11 weeks, and 11 patients (73%) remained dysphagia-free until death. They concluded that a short course of radiotherapy plus chemotherapy might produce complete relief of swallowing difficulties in a substantial proportion of patients with acceptable toxicity. In our study, the overall symptom control rate and one-year symptom control rate were 64% and 61.3%, respectively, in CCRT and 52% and 33.2% in RT alone. There were no severe complications in the CCRT group.

Our study has some limitations. First, this study was retrospectively analysis. It had heterogeneous patient's group and radiation dose. The results may be affected by the selection biases. Second, this study was performed in a small sample size. To conclude the effectiveness of the higher BED≥ 40 Gy_10 _and CCRT to symptomatic pelvic recurrence, prospective randomized trial might be needed.

## Conclusions

In patients with a pelvic mass combined with distant metastasis, symptom palliation was achieved in 80% of the cases, and the median symptom control duration was five months. For improvement of symptom control rate and duration, a higher BED ≥ 40 Gy_10 _is recommended when possible. Considering the low morbidity with CCRT and improved symptom palliation, CCRT might be considered in patients with good performance status.

## Competing interests

The authors declare that they have no competing interests.

## Authors' contributions

WP made contribution to conception and design of the study, and revised the manuscript. SHB contributed to acquisition of data, analysis and interpretation of data, and drafted the manuscript. DHC and HN helped in literature research and revision of the manuscript. YSP, JOP, WYL, SHY and HCK participated in design of study. WKK and HKC gave some intellectual recommendation. All authors have read and approved the final manuscript.
